# Phosphorylation of eukaryotic initiation factor-2α (eIF2α) in autophagy

**DOI:** 10.1038/s41419-020-2642-6

**Published:** 2020-06-08

**Authors:** Juliette Humeau, Marion Leduc, Giulia Cerrato, Friedemann Loos, Oliver Kepp, Guido Kroemer

**Affiliations:** 1 Equipe Labellisée par la Ligue Contre le Cancer, Université de Paris, Sorbonne Université, INSERM UMR1138, Centre de Recherche des Cordeliers, Paris, France; 2 Metabolomics and Cell Biology Platforms, Gustave Roussy, Villejuif, France; 3 Faculty of Medicine, Université Paris Sud, Paris Saclay, Kremlin Bicêtre, France; 4 Suzhou Institute for Systems Medicine, Chinese Academy of Medical Sciences, Suzhou, China; 5 Pôle de Biologie, Hôpital Européen Georges Pompidou, AP-HP, Paris, France; 6 Department of Women’s and Children’s Health, Karolinska University Hospital, Karolinska Institutet, Stockholm, Sweden

**Keywords:** Macroautophagy, Cell signalling

## Abstract

The integrated stress response is characterized by the phosphorylation of eukaryotic initiation factor-2α (eIF2α) on serine 51 by one out of four specific kinases (EIF2AK1 to 4). Here we provide three series of evidence suggesting that macroautophagy (to which we refer to as autophagy) induced by a variety of distinct pharmacological agents generally requires this phosphorylation event. First, the induction of autophagic puncta by various distinct compounds was accompanied by eIF2α phosphorylation on serine 51. Second, the modulation of autophagy by >30 chemically unrelated agents was partially inhibited in cells expressing a non-phosphorylable (S51A) mutant of eIF2α or lacking all four eIF2α kinases, although distinct kinases were involved in the response to different autophagy inducers. Third, inhibition of eIF2α phosphatases was sufficient to stimulate autophagy. In synthesis, it appears that eIF2α phosphorylation is a central event for the stimulation of autophagy.

## Introduction

The so-called integrated stress response^1–3^ is characterized by the phosphorylation of eukaryotic initiation factor-2α (eIF2α). The phosphorylation of eIF2α occurs on serine 51 in response to the activation of one out of four eIF2α kinases^4–6^. EIF2AK1, commonly known as heme-regulated inhibitor (HRI), is activated by oxidative, osmotic, and heat stress, as well as by arsenic and redaporfin-mediated photodynamic therapy^7–10^. EIF2AK2, commonly known as protein kinase R (PKR), is activated by viruses and alcohol^11,12^. EIF2AK3, commonly known as protein kinase R-like endoplasmic reticulum (ER) kinase (PERK), is activated by ER stress, because unfolded proteins in the ER lumen occupy the chaperone GRP78, which then releases PERK from inhibition^13,14^. EIF2AK4, commonly known as general control nonderepressible 2 (GCN2), is activated by nutrient deprivation^15,16^. Thus a variety of rather distinct stressors converge on eIF2α phosphorylation (peIF2α).

peIF2α causes a block in cap-dependent translation, thus reducing general protein synthesis while favoring that of cap-independent, often internal ribosome entry site-dependent proteins^17^. This results in a global shift of translational programs, favoring, for example, the (cap-independent) synthesis of activating transcription factor 4 (ATF4), which then translocates to the nucleus to transactivate a transcriptional program^18^, allowing cells to adapt to stress, for example, by enhancing the expression of chaperones (which help refolding misfolded proteins), by stimulating the removal of unfolded proteins by endoplasmic reticulum associated protein degradation (ERAD), by activating autophagy^19,20^, which is the most efficient pathway for the removal of damaged organelles, or by igniting apoptotic pathways for whole-cell removal when stress is chronic and cannot be overcome. Thus peIF2α indeed stimulates a coordinated ensemble of stress responses that provide cytoprotection when the intensity and duration of the stress is limited, yet allows for the controlled elimination of cells that are irreversibly or chronically damaged^21^.

The central role of peIF2α for autophagy induction was discovered by Beth Levine’s group in the context of starvation of yeast or mouse cells^20^. Since autophagy is (one of) the most important stress-adaptive mechanisms and even determines organismal longevity^22,23^, we wondered whether autophagy would generally rely on peIF2α to be induced. Here we performed a systematic analysis of mammalian cells responding to a vast panel of autophagy modulators to determine their dependence on peIF2α and eIF2α kinases.

## Results and discussion

### Correlation of peIF2α and autophagy

**Fig. 1:**
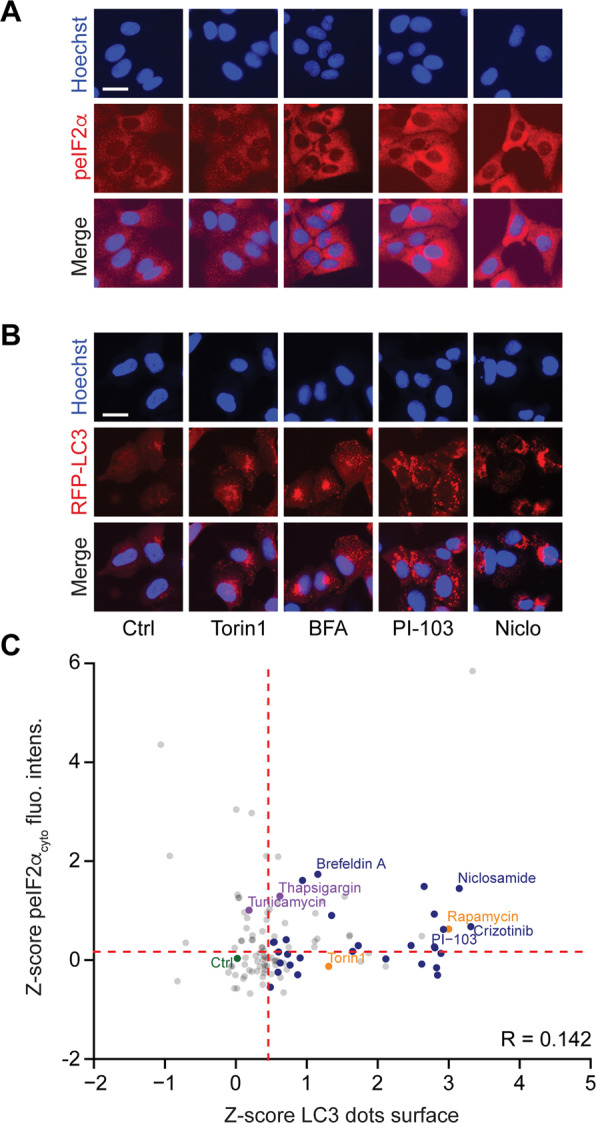
EIF2α phosphorylation and autophagy in U2OS cells. Human osteosarcoma U2OS cells stably expressing GFP-LC3 were treated with the custom arrayed library of autophagy-modulating agents and controls for 6 h. After fixation, the cells were stained with a phosphoneoepitope-specific eIF2α antibody followed by an AlexaFluor-568 secondary antibody. Nuclei were counterstained with Hoechst 33342, and phosphorylation was assessed by fluorescence microscopy (**a**). U2OS RFP-LC3 cells were treated as described above. After fixation and staining with Hoechst 33342, images were acquired (**b**). Representative images are shown for control (Ctrl), torin 1, brefeldin A (BFA), niclosamide (Niclo), and PI-103 (**a**, **b**). For the assessment of autophagy, the average surface of RFP-LC3 dots per cell was quantified. The phosphorylation of eIF2α was evaluated by measuring the cytoplasmic fluorescence intensity of the immunostaining. Data were subjected to a *z*-score transformation centered on control. For LC3 dot surface, the mean of technical quadruplicates from one experiment is shown. For cytoplasmic peIF2α fluorescence intensity, the mean of three independent experiments is depicted. The correlation (*R*) between LC3 dot surface and peIF2α fluorescence intensity was calculated employing Spearman’s rank test. A threshold to select autophagy- and peIF2α-inducing agents was established based on the 60% quartile and distribution, respectively (**c**).

**Fig. 2:**
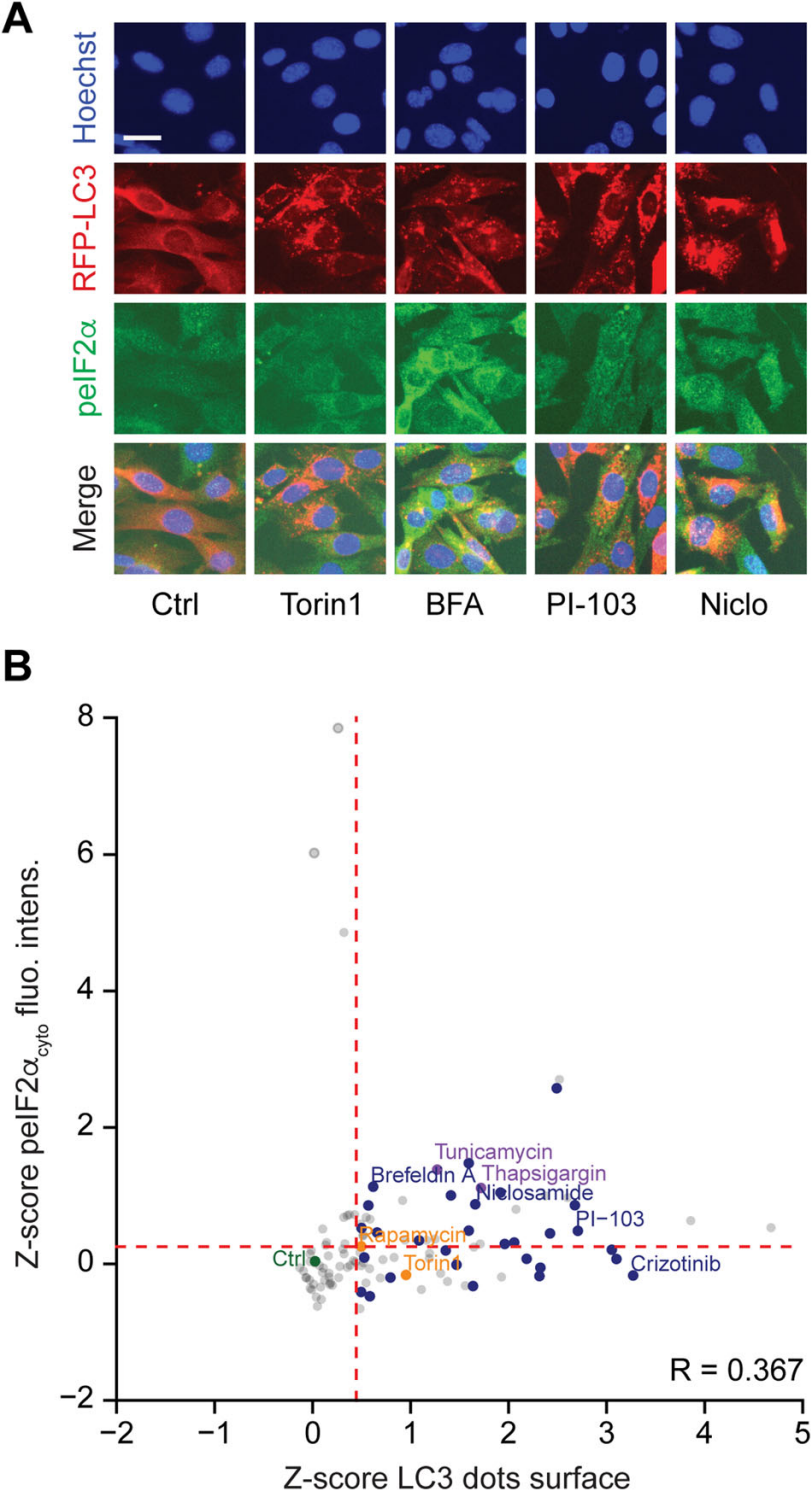
EIF2α phosphorylation and autophagy in MEF cells. Mouse embryonic fibroblasts (MEFs) stably expressing RFP-LC3 were treated with the custom arrayed library of autophagy-modulating agents and controls for 6 h. After fixation, the cells were stained with a phosphoneoepitope-specific eIF2α antibody followed by an AlexaFluor-488 secondary antibody. Nuclei were counterstained with Hoechst 33342 and LC3 aggregation as well as eIF2α phosphorylation were assessed by fluorescence microscopy. Representative images are shown for control (Ctrl), torin 1, brefeldin A (BFA), niclosamide (Niclo), and PI-103 (**a**). The cytoplasmic fluorescence intensity of the immunostaining and the average surface of RFP-LC3 dots per cell were quantified. They were subjected to a *z*-score transformation centered on control. The mean of technical quadruplicates is shown. The correlation (*R*) between LC3 dot surface and peIF2α fluorescence intensity was calculated using Spearman’s rank test. A threshold to select autophagy- and peIF2α-inducing agents was established based on the 60% quartile and distribution, respectively (**b**).

As a first approach to investigate the interdependence between autophagy and peIF2α, we measured the phosphorylation of eIF2α in human osteosarcoma U2OS cells by means of an immunofluorescence staining protocol with a phosphoneoepitope-specific antibody recognizing eIF2α phosphorylated on serine 51 (Figs. S1 and 1a). We also quantified autophagic puncta in cells stably transfected with red fluorescent protein (RFP) fused to microtubule-associated proteins 1A/1B light chain 3B (hereafter referred to as LC3) by fluorescence microscopy (Fig. 1b). We took advantage of the Enzo library of autophagy modulators (all used at 10 µM) that was complemented by a series of caloric restriction mimetics including several polyamines and chalcones^24–26^, several microtubule inhibitors^27^ as well as crizotinib^26,28^, all of which were added at doses that are expected to stimulate autophagy. As shown in Fig. 1c, many of the strong inducers of autophagic puncta also stimulated peIF2α. Similar results were obtained in mouse embryonic fibroblasts (MEFs) exposed to the same collection of compounds (Fig. 2a, b). Of note, in both U2OS and MEF cells, torin 1, which is a direct inhibitor of mechanistic target of rapamycin (mTOR), induced autophagy without enhancing peIF2α, in line with a prior report^29^. If the intensity of peIF2α and the surface of RFP-LC3 dots were measured on a cell-per-cell basis in U2OS, the two parameters were found to correlate among each other, both in baseline condition and after pharmacological autophagy induction (Fig. S2). Thus induction of LC3 puncta is often, but not always, coupled to peIF2α.

### Dependency of autophagy on peIF2α

**Fig. 3:**
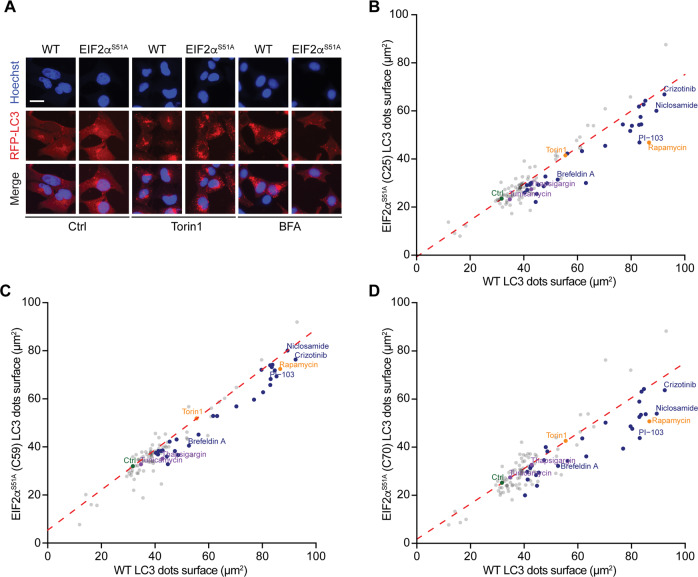
Role of eIF2α phosphorylation for autophagy modulation in U2OS cells. Human osteosarcoma U2OS cells stably expressing RFP-LC3 wild type (WT) and knockin for *EIF2a*^*S51A*^ (clones 25, 59, and 70, respectively, abbreviated as C25, C59, and C70) were treated with the custom arrayed library of autophagy-modulating agents and controls for 6 h. After fixation, nuclei were counterstained with Hoechst 33342. Representative images are shown for control (Ctrl), torin 1, and brefeldin A (BFA) in WT and C70 cells (**a**). RFP-LC3 dot surface was quantified and the mean of technical quadruplicates from one experiment was plotted for *EIF2a*^*S51A*^ knockin clones 25 (**b**), 59 (**c**), and 70 (**d**) versus the WT cell line. A linear regression was performed for control and torin 1 (which induces autophagy independent of eIF2α phosphorylation) (**b**–**d**).

**Fig. 4:**
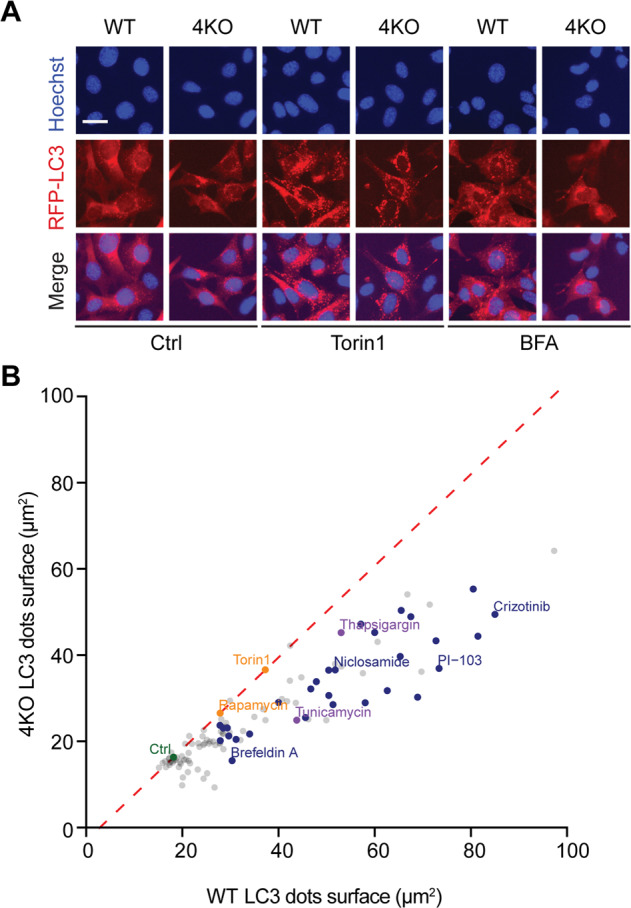
Role of eIF2α phosphorylation for autophagy modulation in MEF cells. Mouse embryonic fibroblasts (MEFs) stably expressing RFP-LC3 wild type (WT) and knockout for *eif2ak*1–4 (*4KO*) were treated with the custom arrayed library of autophagy-modulating agents and controls for 6 h. After fixation, nuclei were counterstained with Hoechst 33342. Representative images are shown for control (Ctrl), torin 1, and brefeldin A (BFA) in WT and 4KO cells (**a**). RFP-LC3 dot surface was quantified and the mean of technical quadruplicates was plotted for the *4KO* cell line versus the WT cell line. A linear regression was performed for control and torin 1 (which induces autophagy independent of eIF2α phosphorylation) (**b**).

**Fig. 5:**
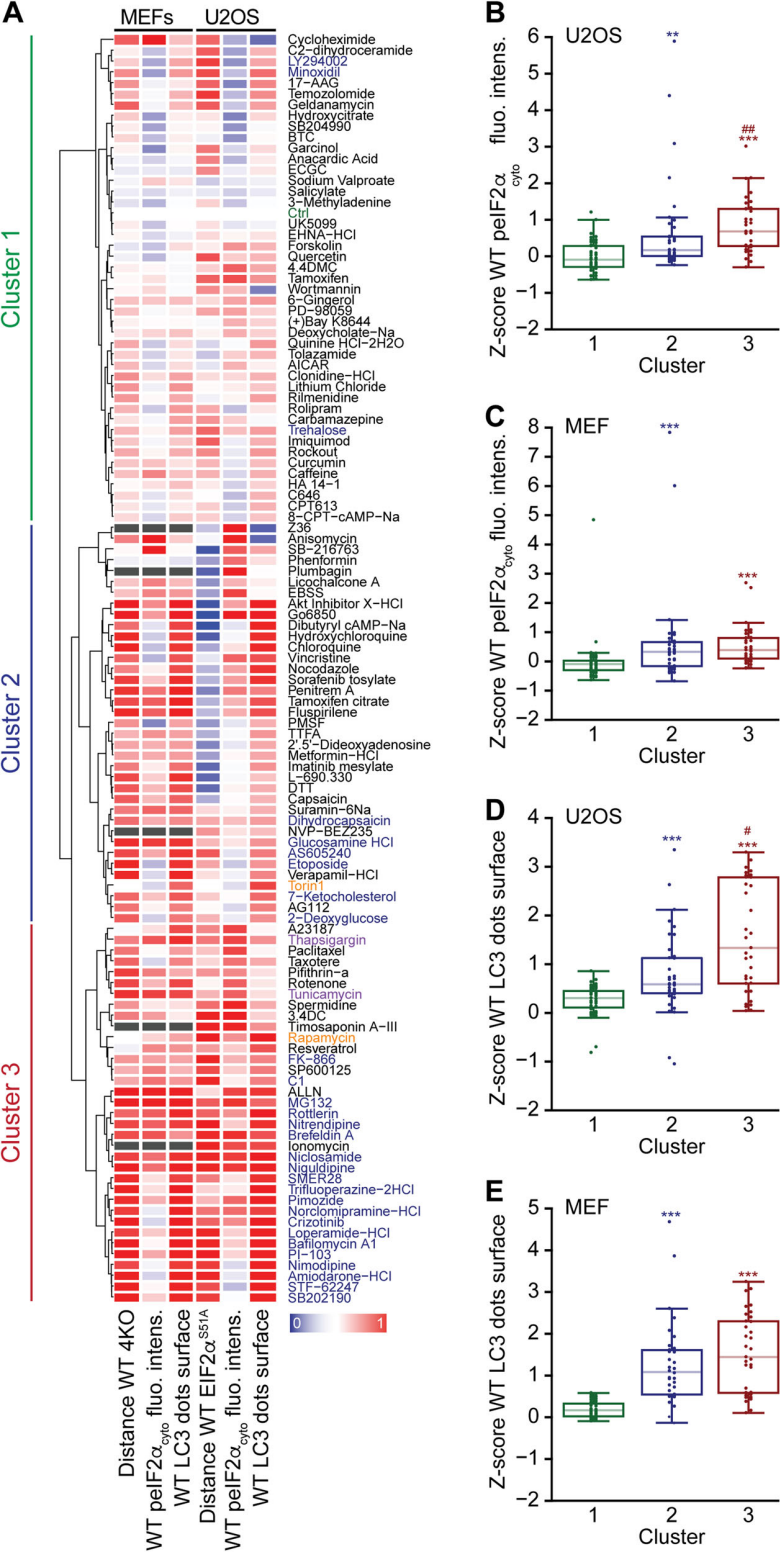
eIF2α phosphorylation participates in the induction of autophagy in certain contexts. The heatmap summarizes the effects of the agents of the custom arrayed library of autophagy modulators in MEF and U2OS on LC3 dot surface and peIF2α in addition to the dependency of LC3 dot surface on eIF2α phosphorylation. Agents that caused toxicity in both cell lines were excluded from the analysis and marked with gray color when toxic in only one cell line. The geometric distances of each point in LC3 dot surface between U2OS WT and *EIF2a*^*S51A*^ (from Fig. 3b–d) as well as between MEF WT and knockout for *eif2ak1–4* (*4KO*) (Fig. 4b) were calculated. It reflects the dependency of autophagy induction on eIF2α phosphorylation, with a positive distance allocated to points located under the regression curve (corresponding to agents that requires peIF2α for complete autophagy induction). In U2OS, the mean of the distances of the three tested clones was calculated. Distances were subjected to a *z*-score transformation centered on control. Then the *z*-score of LC3 dot surface and peIF2α cytoplasmic fluorescence intensity (from Fig. 1c for U2OS and Fig. 2b for MEF) as well as geometric distances were independently scaled between 0 and 1 with a sigmoidal transformation and represented. Agents that are among the 40% most potent autophagy inducers and for which autophagy depends on peIF2α (with a distance >0.5) in both cell lines are shown in dark blue. In orange positive controls for autophagy and in purple positive controls for peIF2α are depicted. Hierarchical clustering was performed, leading to three main clusters (**a**). For agents in each cluster (1, 2 and 3), *z*-scores of peIF2α in U2OS (**b**) and in MEFs (**c**) as well as of LC3 dot surface in U2OS (**d**) and in MEFs (**e**) are shown as boxplots with median ± quartiles ± 95% confidence intervals. Statistical significance was analyzed using a Kolmogorov–Smirnov test. Differences to cluster 1 are depicted as **p < 0.01 and ***p < 0.001 and differences between clusters 2 and 3 as ^#^*p* < 0.05 and ^##^*p* < 0.01 (**b**–**e**).

In order to understand to which extent peIF2α is required for autophagy induction, U2OS cells stably transduced with RFP-LC3 were subjected to CRISPR-Cas9-driven mutagenesis to render eIF2α non-phosphorylable (due to the exchange of serine 51 by an alanine residue yielding EIF2α^S51A^). We validated three *EIF2*α^*S51A*^ homozygous clones (C25, C59, and C70) by genomic sequencing and immunofluorescence analysis (Fig. S3). Of note, all the three clones exhibited reduced RFP-LC3 puncta in response to many of the pharmacological autophagy modulators with the notable exception of torin 1 (Fig. 3). As a complementary approach, we used MEFs that had been subjected to the knockout of all eIF2α kinases (EIF2AK1 commonly known as HRI, EIF2AK2 commonly known as PKR, EIF2AK3 commonly known as PERK, EIF2AK4 commonly known as GCN2)^5^, finding again that most pharmacological autophagy enhancers display a lower pro-autophagic potential in the absence of peIF2α (Fig. 4). When all results were combined and subjected to hierarchical clustering, three major clusters (1–3) emerged (Fig. 5a). Of note, the strongest inducers of autophagic puncta that depended in their activity on both phosphorylable eIF2α and the eIF2α kinases (cluster 3) exhibited a significantly higher phosphorylation level of eIF2α than the weak autophagy inducers (cluster 1) (Fig. 5b–d).

**Fig. 6:**
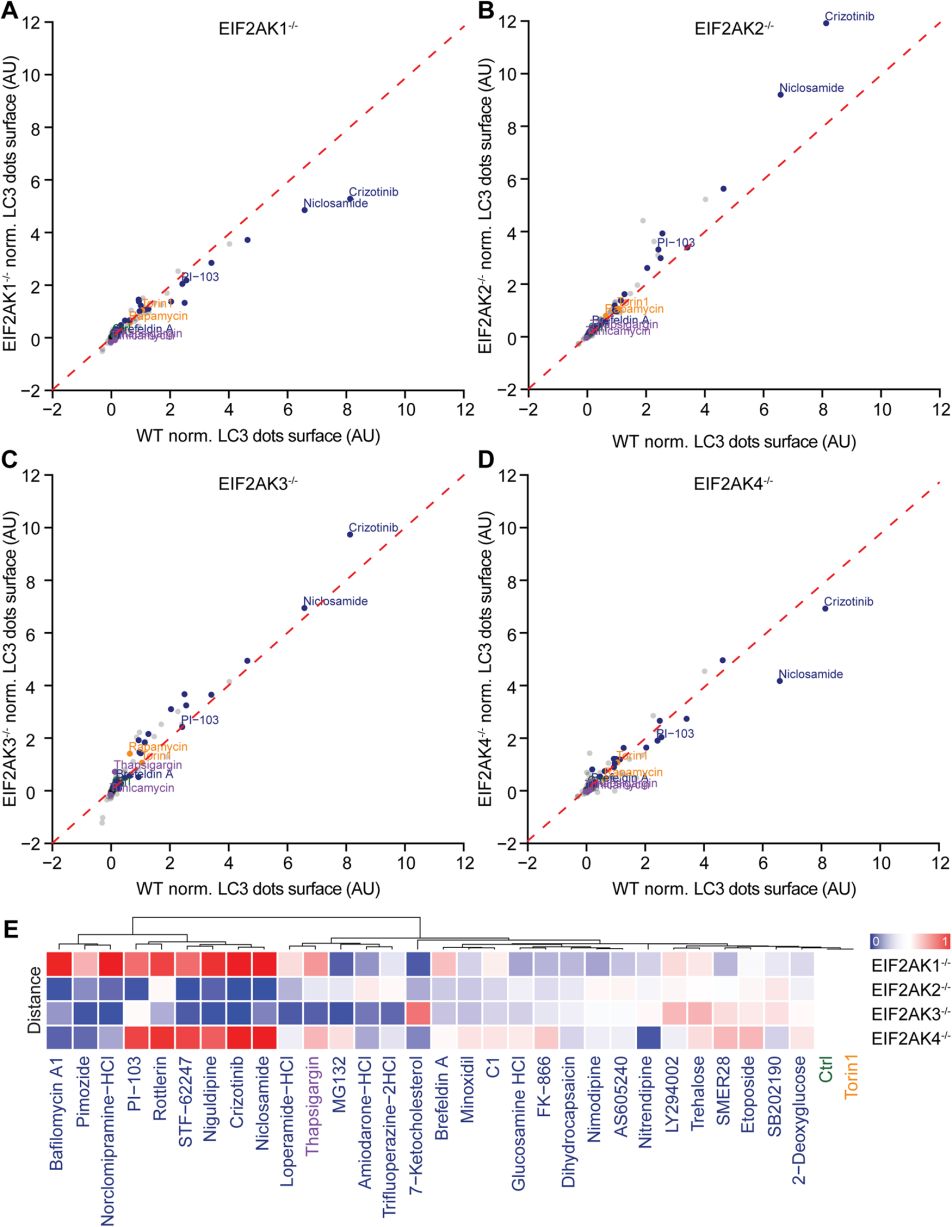
Role of the eIF2α kinases 1–4 in autophagy. Human osteosarcoma U2OS cells stably expressing GFP-LC3 either wild type (WT) or knockout for *EIF2AK1*, *2*, *3*, *4* were treated with the custom arrayed library of autophagy-modulating agents and controls for 6 h. After fixation, nuclei were counterstained with Hoechst 33342 (**a**). The samples were acquired by fluorescence microscopy and LC3 dot surface was quantified. The data were normalized as percentage of induction with the untreated condition as negative control and torin 1 as positive control. The mean of LC3 dot surface from three independent experiments was plotted for *EIF2AK1*^−*/*−^ (**a**), *EIF2AK2*^−*/*−^ (**b**), *EIF2AK3*^−*/*−^ (**c**), and *EIF2AK4*^−*/*−^ (**d**) versus the WT cell line. A linear regression was performed for control and torin 1 in each *EIF2AK*^*−/−*^ cell line to the WT. For the agents that were identified as requiring eIF2α phosphorylation for complete autophagy induction in Fig. 5, the geometric distances to the linear regression were calculated, transformed to *z*-scores, independently scaled between 0 and 1 with a sigmoidal transformation, and represented (**e**).

We also measured the contribution of each individual eIF2α kinase to autophagy by knocking them out individually in U2OS cells stably expressing green fluorescent protein (GFP)-LC3 (Fig. S4). For this, we concentrated on those pharmacological agents that were the strongest inducers of RFP-LC3 puncta and tended to depend in their activity on peIF2α, contrasting with torin 1 (Fig. S5). For these compounds, the knockout of EIF2AK1 and EIF2AK4 had the highest autophagy-inhibitory impact, although EIF2AK3 appeared to be relevant for some autophagy inducers as well (Fig. 6). Altogether, these results suggest that distinct pharmacological autophagy enhancers rely on different eIF2α kinases to be efficient and that some functional redundancy among such kinases exists.

### Autophagy induction by eIF2α phosphatase inhibitors

**Fig. 7:**
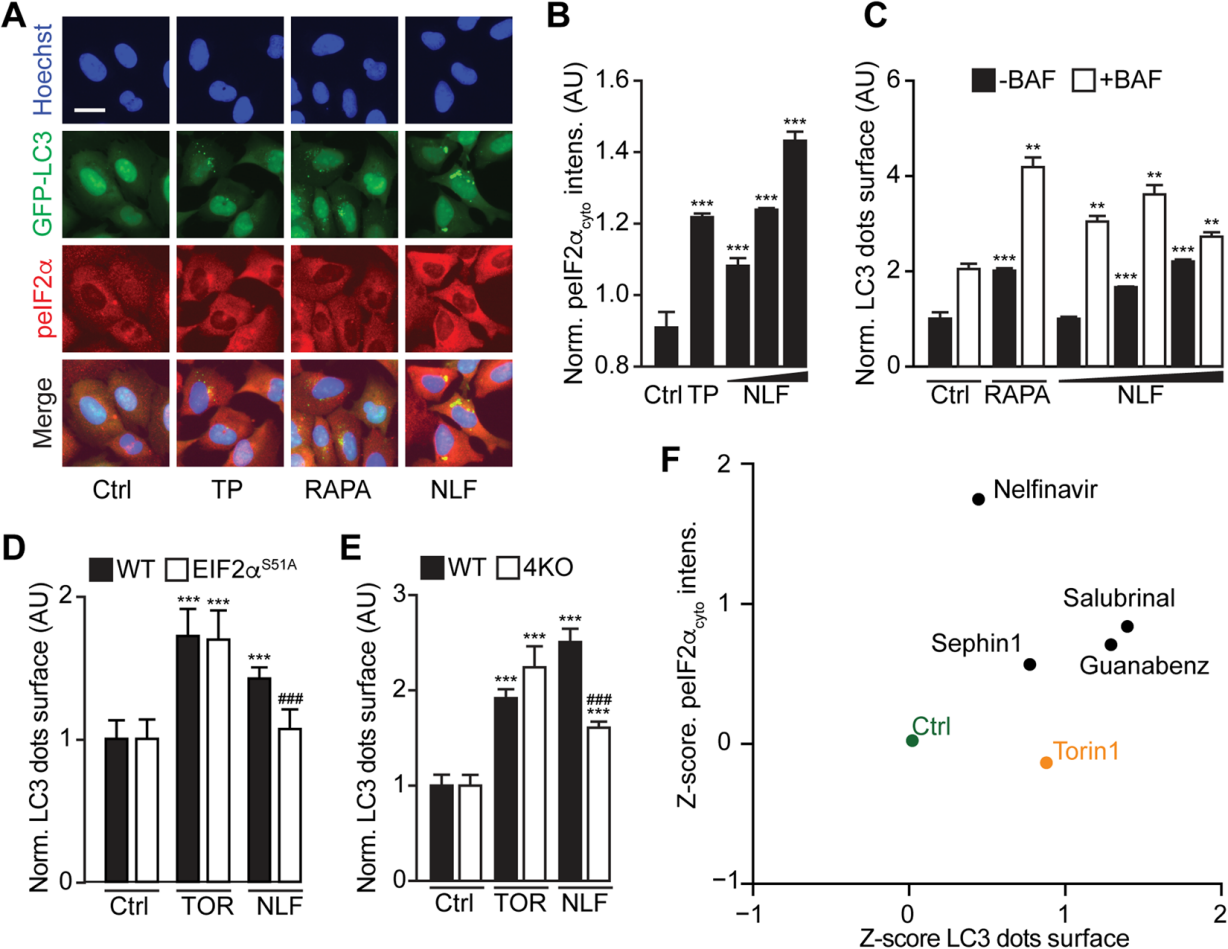
Autophagy induced by eIF2α phosphatase inhibitors. Human osteosarcoma U2OS cells stably expressing GFP-LC3 were treated with nelfinavir (NLF) at 10, 20, and 40 μM for 6 h. Rapamycin (RAPA) at 20 μM was used as a positive control for autophagy induction and thapsigargin (TP) at 3 μM was used as a positive control for eIF2α phosphorylation. The treatments were performed in the presence and absence of the lysosomal fusion inhibitor bafilomycin A1 (BAF). For this, BAF at 0.1 μM was supplemented after the first 4 h of treatment. Following fixation, the cells were stained with a phosphoneoepitope-specific eIF2α antibody followed by an AlexaFluor-568 secondary antibody. Nuclei were counterstained with Hoechst 33342, and autophagy and phosphorylation of eIF2α were assessed by fluorescence microscopy (**a**–**c**). The different conditions were normalized as percent of control (Ctrl). Representative images and mean ± SD of triplicates (among four replicates, the one that had the highest deviation from the mean was excluded) from one representative experiment among three is shown. Statistical significance was analyzed using a Student’s *t* test. Differences to respective controls (with or without BAF) are depicted as **p < 0.01 and ***p < 0.001 (**b**, **c**). U2OS RFP-LC3 WT and three clones of U2OS knockin for *EIF2a*^*S51A*^ were treated with torin 1 (TOR) at 300 nM as a positive control and nelfinavir (NLF) at 40 mM for 6 h. LC3 dot surface was measured and normalized as percent of Ctrl. Depicted are mean ± SD of quadruplicates and statistical significance was analyzed using Student’s *t* test. Differences to controls are depicted as ***p < 0.001 in the same cell line and as ^###^*p* < 0.001 between WT and *EIF2a*^*S51A*^ clones (**d**). Mouse embryonic fibroblasts (MEFs) stably expressing RFP-LC3 either WT or knockout for *eif2ak1–4* (*4KO*) were treated with torin 1 (TOR) at 300 nM as a positive control and nelfinavir (NLF) at 40 mM for 6 h. After fixation, the cells were stained with a phosphoneoepitope-specific eIF2α antibody followed by an AlexaFluor-488 secondary antibody. Nuclei were counterstained with Hoechst 33342 and autophagy and phosphorylation were assessed by fluorescence microscopy. LC3 dots surfaces for each condition were normalized as percent of their respective Ctrl in the same cell line. Depicted are mean ± SD of quadruplicates and statistical significance was analyzed using the Student’s*-*test. Differences to controls are depicted as ***p < 0.001 in the same cell line and as ^###^*p* < 0.001 between WT and *4KO* (**e**). U2OS GFP-LC3 were treated with nelfinavir at 40 mM, sephin 1 at 50 mM, salubrinal at 80 mM, guanabenz at 50 mM and torin 1 at 300 nM as a positive control for 6 h. After fixation, the cells were stained with a phosphoneoepitope-specific eIF2α antibody followed by an AlexaFluor-568 secondary antibody. Nuclei were counterstained with Hoechst 33342 and autophagy and phosphorylation were assessed by fluorescence microscopy. LC3 dot surface and peIF2α fluorescence intensity in the cytoplasm were measured, submitted to a *z*-score transformation and depicted (**f**).

We next addressed the question whether the stimulation of peIF2α might be sufficient for the induction of autophagy. For this, we took advantage of nelfinavir, an inhibitor of the phosphatase that constitutively dephosphorylates eIF2α^30,31^. Nelfinavir did not only cause the hyperphosphorylation of eIF2α but also stimulated the generation of GFP-LC3 puncta (Fig. 7a–c). Of note, nelfinavir combined with bafilomycin A1, an inhibitor of vacuolar-type H^+^-ATPase and hence an inhibitor of the final stage of autophagy^32^, induced more GFP-LC3 puncta than in cells treated with bafilomycin A1 alone, a finding indicating that nelfinavir stimulates autophagic flux. The induction of GFP-LC3 puncta depended on the phosphorylability of eIF2α (because the effect was lost in EIF2α^S51A^ cells) and the activity of eIF2α kinases (because the effect was suppressed in cells lacking the four eIF2α kinases) (Fig. 7d, e). Importantly, a variety of additional inhibitors of eIF2α dephosphorylation (such as guanabenz, salubrinal, and sephin 1) shared the capacity of nelfinavir to simultaneously stimulate peIF2α and autophagy (Fig. 7f).

### Concluding remarks

As mentioned in the “Introduction” section, peIF2α is part of the integrated stress response, which is connected to autophagy. However, a systematic study of the requirement of peIF2α and the involvement of distinct eIF2α kinases has been elusive. Here we used a collection of approximately 200 compounds to show that most inducers of LC3 puncta require peIF2α and eIF2α kinases to be efficient. There are three major arguments that favor the hypothesis that peIF2α is important for the activation of autophagy: (i) the stimulation of autophagic puncta and peIF2α correlates for many autophagy modulators; (ii) cells bearing a non-phosphorylable eIF2α mutant or lacking all known eIF2α kinases are refractory to autophagy induction by most stimuli; and (iii) activation of peIF2α by inhibition of a specific set of phosphatases suffices to trigger autophagy.

In accord with previous studies, the proximal mode of action of distinct autophagy enhancers may depend on distinct eIF2α kinases. Thus EIF2AK4 (GCN2) is important for autophagy induction by nutrient deprivation^15,16^, contrasting with the fact that primary ER stress mediated by, for example, polyglutamine repeats, tends to require EIF2AK3 (PERK)^33^ and saturated fatty acids (such as palmitate) rely on EIF2AK2 (PKR) to stimulate autophagic puncta. This latter pathway is complex, requiring the phosphorylation of heat shock protein 27, resulting in its binding to STAT3, which then releases EIF2AK2 from its inhibition to facilitate peIF2α and autophagy induction^34,35^. Thus rather different proximal stimuli can converge on distinct eIF2α kinases to trigger a similar integrated stress response leading to autophagy.

It should be noted that peIF2α is not only important for autophagy induction but also contributes to a variety of stress pathways including the induction of ER stress^36^, the formation of stress granules^37^, and the translocation of calreticulin to the surface of stressed cells where it acts as “eat me” signal for the removal of the stressed cells by phagocytosis^38^. This latter phenomenon is highly important for the dendritic cell-dependent immune recognition of cancer cells in the context of “immunogenic cell death”^39^ meaning that peIF2α is actually a central hallmark of this cell death modality^27^. Thus peIF2α can be viewed as a central hub that signals in favor of immune responses, that minimizes the replication of intracellular pathogens (through inhibition of protein synthesis in the ER), sequesters and eliminates them in the cytoplasm (through autophagy/xenophagy), facilitates the destruction of infected cells (through phagocytosis/phagoptosis), or connects them to T lymphocyte-mediated immune recognition (subsequent to antigen cross-presentation by dendritic cells). Importantly, viruses may encode proteins that either inhibit eIF2α kinases or dephosphorylate eIF2α to inhibit peIF2α and to escape from cell-autonomous and immune recognition^40–42^. From this point of view, it appears intriguing that prominent antiretroviral agents such as nelfinavir, which is usually considered as a HIV-1 inhibitor^30^, may have off-target effects that favor peIF2α and hence may stimulate a broad immune response. Future work must determine to what extent this effect may contribute to the clinical efficacy of nelfinavir and similar compounds.

## Materials and methods

### Cell lines

Human osteosarcoma U2OS were purchased from the ATCC. U2OS cells stably expressing GFP-LC3 and RFP-LC3 were generated by our group in the past^27,35^. MEF wild type (WT) and knockout for the four eIF2α kinases (MEF *4KO*) were provided by Professor Seiichi Oyadomari from Tokushima University. MEF WT RFP-LC3 and *4KO* RFP-LC3 were constructed from the aforementioned cell lines, which were transduced with LentiBright lentiviral particles coding for RFP-LC3 (17–10143, Millipore, Burlington, MO, USA). The day following transduction, cells were supplemented with fresh medium and, 1 day later, single cell sorted by flow cytometry based on RFP fluorescence. U2OS cells stably expressing RFP-LC3 bearing a mutant non-phosphorylable version of eIF2α (EIF2α^S51A^) were constructed using the CRISPR-Cas9 technology as previously detailed^43^. Briefly, we designed a guide RNA (gRNA) targeting eIF2α and inserted them into the pX458 vector (containing a tracrRNA and Cas9 fused with 2A-GFP)^44^ following the manufacturer’s protocol (New England Biolabs, Ipswich, MA, USA). This plasmid was used together with a homology repair template that targets serine in position 51 of eIF2α for an exchange to alanine to transfect RFP-LC3 expressing U2OS cells by means of Lipofectamine 2000 (Thermo Fisher Scientific, Waltham, MA, USA) according to the manufacturer’s protocol. Two days later, single cells were sorted by flow cytometry. DNA of clones that grew was extracted, amplified by PCR, and analyzed for homozygous knockin by sequencing (Eurofins Scientific, Luxembourg). They were further validated by immunofluorescence (Fig. S3). U2OS GFP-LC3 having one eIF2α kinase knocked out (*EIF2AK1*^−*/*−^, *EIF2AK2*^−*/*−^, *EIF2AK3*^−*/*−^, and *EIF2AK4*^−*/*−^) were constructed using an U6gRNA-Cas9-2A-RFP plasmid containing gRNAs (Sigma-Aldrich, St. Louis, MO, USA) following the manufacturer’s protocol as previously described^43^. In short, U2OS GFP-LC3 cells were transfected, and 2 days later, single cells were sorted by flow cytometry. Clones were validated by immunoblot with specific antibodies against human EIF2AK1 (HRI), EIF2AK2 (PKR), EIF2AK3 (PERK), and EIF2AK4 (GCN2) (Fig. S4A) and by immunofluorescence (Fig. S4B).

### Cell culture

Human osteosarcoma U2OS cells and MEF cells were cultured in Dulbecco’s modified Eagle’s medium (Thermo Fisher Scientific) supplemented with 10% fetal bovine serum (Gibco by Thermo Fisher Scientific), 1% non-essential amino acids (Thermo Fisher Scientific), and 1% HEPES (4-(2-hydroxyethyl)-1-piperazineethanesulfonic acid; Thermo Fisher Scientific) in a humidified incubator with 5% CO_2_ at 37 °C. Cell culture plastics and consumables were purchased from Greiner Bio-One (Kremsmünster, Austria) or Corning (NY, USA).

### Antibodies

Rabbit monoclonal phosphoneoepitope-specific antibody against phospho-eIF2α (Ser51) (ab32157) (used with U2OS cells) and mouse monoclonal antibody against β-actin (ab49900) were purchased from Abcam (Cambridge, UK). Rabbit polyclonal antibody against HRI (sc-30143) and mouse monoclonal antibody against PKR (sc-6282) were purchased from Santa Cruz biotechnology (Dallas, TX, USA). Rabbit mouse monoclonal phosphoneoepitope-specific antibody against phospho-eIF2α (Ser51) used with MEF cells (#3597), rabbit monoclonal antibody against PERK (#3192), and rabbit polyclonal antibody against GCN2 (#3302) came from Cell Signaling Technology (Danvers, MA, USA). Anti-rabbit and anti-mouse AlexaFluor-488, -568, and -647 secondary antibodies came from Thermo Fisher Scientific.

### Compounds

The Enzo autophagy library came from Enzo Life Science (Farmingdale, NY, USA). In addition, crizotinib (PZ0191), docetaxel (01885), doxorubicin (D1515), paclitaxel (T7191), rapamycin (R0395), thapsigargin (T9033), tunicamycin (T7765), vinblastine sulfate (V1377), vincristine sulfate (V0400000), 3,4‐dimethoxychalcone abbreviated as 3.4DC (S798126), 4,4′‐dimethoxychalcone abbreviated as 4.4 DMC (S617237), sodium arsenate dibasic hepta-hydrate (A6756), spermidine (740780), anacardic acid (A7236), C646 (SML0002), quercetin hydrate (337951), sodium salicylate (S3007), potassium hydroxycitrate tribasic monohydrate (59847), phenformin hydrochloride (SC219590), 1-cyano-4-dimethylaminopyridinium tetrafluoroborate abbreviated as CPT613 (C2776), UK5099 (PZ0160), epigallocatechingallate (E4143), curcumin (C1386), resveratrol (R5010), 1,3,5-benzenetricarboxylic acid abbreviated as BTC (8012990025), nelfinavir mesylate hydrate (PZ0013), sephin1 (SML1356), guanabenz acetate (G110), and salubrinal (SML0951) have been bought from Sigma-Aldrich. Garcinol (BML-GR343) have been purchased from Enzo Life Science. SB204990 (4962) and torin 1 (4247) have been purchased from Tocris (Bristol, UK).

A custom arrayed library used for various experiments in the study (Figs. 1–6 and S5) was made from the compounds of the Enzo autophagy library at 10 μM (except for bafilomycin A1, which was used at 1 μM) supplemented with 3,4‐dimethoxychalcone at 30 μM, 4,4′‐dimethoxychalcone at 50 μM, hydroxycitrate at 10 mM, phenformin at 3 mM, salicylate at 5 mM, spermidine at 100 μM, anacardic acid at 50 μM, C646 at 10 μM, epigallocatechingallate at 50 μM, CPT613 at 100 μM, curcumin at 50 μM, resveratrol at 50 μM, SB204990 at 100 μM, benzenethicarxylic acid at 5 mM, UK5099 at 1 μM, garcinol at 10 μM, quercetin at 50 μM, taxotere at 3 μM, paclitaxel at 3 μM, vincristine at 3 μM, crizotinib at 15 μM, torin 1 at 0.3 μM, and rapamycin at 20 μM. Staurosporine and SU1152 were excluded from the analysis because of their toxicity that induced a phenotype preventing a relevant analysis of the LC3 aggregation.

Tunicamycin and thapsigargin at 3 or 10 μM were used all along the study as positive controls for peIF2α and are written in purple in the figures. Rapamycin at 10 μM and torin 1 at 0.3 μM were used as positive controls for autophagy and are written in orange, with torin 1 as peIF2α-independent autophagy inducer. The hits that are shown in Figs. 5 and S5 as peIF2α-dependent autophagy inducers are depicted in dark blue in all the figures. Untreated control is shown in green.

### Fluorescence microscopy, image acquisition, and analysis

One day prior to treatment, 2500 U2OS cells (RFP-LC3 WT or *EIF2*α^*S51A*^, GFP-LC3 WT, *EIF2AK1*^−*/*−^, *EIF2AK2*^−*/*−^, *EIF2AK3*^−*/*−^, or *EIF2AK4*^−*/*−^) or 2000 MEF cells (RFP-LC3 WT or *4KO*) were seeded in 384-well µClear imaging plates (Greiner BioOne) and let to adhere. The next day, cells were treated for 6 h to assess peIF2α and autophagy levels. Following this, cells were fixed with 3.7% formaldehyde (F8775, Sigma Aldrich) supplemented with 1 μg/ml Hoechst 33342 for 1 h at room temperature. When measuring LC3 aggregation for assessing the level of autophagy, the fixative was exchanged to phosphate-buffered saline (PBS), and the plates were analyzed by automated microscopy. peIF2α was assessed by further immunostaining after fixation: unspecific antibody interaction was blocked by 2% bovine serum albumin (BSA) for 1 h at room temperature and followed by incubation with primary antibody overnight at 4 °C. After several washing steps with PBS, cells were stained with AlexaFluor-568 (or 488)-coupled secondary antibody (Thermo Fisher Scientific) for 2 h at room temperature and washed with PBS before acquisition. For automated fluorescence microscopy, a robot-assisted IXM XL BioImager and a IXM-C confocal BioImager (Molecular Devices, Sunnyvale, CA, USA) equipped with either a SpectraX or an Aura II light source (Lumencor, Beaverton, OR, USA), adequate excitation and emission filters (Semrock, Rochester, NY, USA), and a 16-bit monochromes sCMOS PCO.edge 5.5 camera (PCO Kelheim, Germany) or an Andor Zyla camera (Belfast, Northern Ireland) and a 20X PlanAPO objective (Nikon, Tokyo, Japan) were used to acquire a minimum of four view fields per well, followed by automated image processing with the custom module editor within the MetaXpress software (Molecular Devices). The primary region of interest (ROI) was defined by a polygon mask around the nucleus allowing for the enumeration of cells and the detection of morphological alterations of the nucleus and nuclear fluorescence intensity. Secondary cytoplasmic ROIs were used for the quantification of peIF2α intensity. To quantify LC3 aggregation, a mask of high-intensity dots was drawn in the cytoplasm of cells. After exclusion of cellular debris and dead cells from the data set, parameters of interest were normalized, statistically evaluated, and graphically depicted using the R software. Of note, when <30 cells per condition were in good shape, the corresponding condition was excluded from the data set. Using R, images were extracted, and pixel intensities were scaled to be visible (to the same extent for all images of a given experiment). Scale bars represent 20 μm. When needed to compare autophagy among different cell lines, the surface of LC3 dots was normalized as the percentage of induction ((*x* − *c*^+^)/(*c*^−^ − *c*^+^), with *x* test, *c*^+^ positive control torin 1, and *c*^−^ negative control)^45^.

### Method to determine the effect of a genetic modification on the induction of autophagy

In order to compare cell lines, a linear regression of LC3 dot surface (subjected to *z*-score transformation centered on control) between control and torin 1 was performed. Then the geometric distance to this regression was calculated and considered positive when lower in the genetically modified cell line. For plotting heatmaps, data were independently scaled between 0 and 1 with a sigmoidal transformation with control at 0.5. Then hierarchical clustering was performed.

### Protein immunoblot

Protein was extracted with RIPA buffer (#89900; Thermo Fisher Scientific) supplemented with phosphatase and protease inhibitors (#88669; Thermo Fisher Scientific) followed by sonication. Then protein concentration was measured by means of the Bio-rad laboratory DC Protein Assay (#500-0113, #500-0114 and #500-0115, Hercules, CA, USA) following the manufacturer’s protocol. Twenty µg of proteins were resuspended in Laemmli buffer (Thermo Fisher Scientific), denaturated at 100 °C, and separated by means of polyacrylamide gel electrophoresis using 4–12% Bis-Tris pre-casted gels (Thermo Fisher Scientific) in MOPS buffer (Thermo Fisher Scientific). Then proteins were electrotransferred to EtOH-activated polyvinylidene difluoride membranes (Merck Millipore IPVH00010) in transfer buffer (25 mM Tris, 190 mM glycine, 20% methanol in H_2_O) at 200 mA and 120 V for 1.5 h. Membranes were washed in Tris-buffered saline with Tween20 buffer (TBST; 20 mM Tris, pH 7.5 150 mM NaCl 0.1% Tween 20 in H_2_O), and then non-specific sites were blocked with 5% BSA in TBST for 1 h. Membranes were incubated with primary antibody diluted in 5% BSA in TBST overnight at 4 °C. Following this, membranes were washed with TBST and then incubated with appropriate horseradish peroxidase-coupled secondary antibody (Southern Biotech, Birmingham, AL, USA) for 1 h at room temperature. Using ECL (GE Healthcare, Chicago, IL, USA), proteins on the membranes were revealed. Beta-actin was quantified to ensure equal loading.

### Statistical analyses

Statistical analyses were performed using the freely available software R (https://www.r-project.org). To compare the effect of treatments on one biological parameter, data were depicted in barcharts with mean ± SD. The statistical significance was evaluated using one-sided unpaired Student’s *t* test with the *t.test* function from the *stats* R package. Kolmogorov–Smirnov test was used to compare distributions of drugs belonging to different clusters, using the *ks.test* function from the *stats* R package. Such data are depicted as boxplot with median ± quartiles ± 95% confidence intervals. Correlations between two parameters were performed by Spearman’s rank test, using the *ggscatter* function from the *ggpubr* R package.

## Supplementary information

Supplementary Figure Legends

Figure S1. Validation of eIF2αS51 antibody for U2OS cells

Figure S2. Correlation between autophagy and peIF2α in a cell-per-cell basis

Figure S3. Validation of U2OS RFP LC3 EIF2αS51 clones

Figure S4. Validation of U2OS knockout for eIF2α kinases 1, 2, 3 and 4

Figure S5. Agents requiring eIF2α phosphorylation for complete autophagy induction

Table S1

Table S2
