# Supplementary material for: Phosphorylation of eukaryotic initiation factor-2α (eIF2α) in autophagy
Source: Cell Death Dis. 2020 Jun 8;11(6):433. doi: 10.1038/s41419-020-2642-6 (PMC7280501; doi:10.1038/s41419-020-2642-6)
Supplement: Supplementary file 1 — Supplementary Figure Legends [file 41419_2020_2642_MOESM1_ESM.docx]

**Supplementary Figure Legends**

**Figure S1. Validation of eIF2αS51 antibody for U2OS cells**

Human osteosarcoma U2OS cells stably expressing GFP-LC3 were treated with thapsigargin (TP) and tunicamycin (TM) (two compounds well known to induce eIF2α phosphorylation) at 3 μM for 6 h. After fixation, the cells were stained with a phosphoneoepitope-specific eIF2α antibody (ab32157) followed by an AlexaFluor-568 secondary antibody. Nuclei were counterstained with Hoechst 33342 and eIF2α phosphorylation was assessed by fluorescence microscopy. The different conditions were normalized as percent of control (Ctrl). Representative images and mean ± SD of quadruplicates from one representative experiment among three is shown. Statistical significance was analyzed using a Student’s t-test. Differences to controls are depicted as * p < 0.0; ** p < 0.01; *** p < 0.001 (**A, B**). Same conditions were subjected to immunoblotting. After SDS-PAGE, proteins were transferred on a membrane and eIF2α phosphorylated on S51 was detected with a specific antibody as described above, followed by an HRP-coupled secondary antibody. The amount of protein was normalized using β-actin (ACTB). A representative image of one experiment is depicted, as well data as mean ± SD from two independent experiments (**C, D**).

**Figure S2. Correlation between autophagy and peIF2α in a cell-per-cell basis**

Human osteosarcoma U2OS cells stably expressing RFP-LC3 were treated with brefeldin A, PI-103 and niclosamide at 10 μM for 6 h. After fixation, the cells were stained with a phosphoneoepitope-specific eIF2α antibody followed by an AlexaFluor-568 secondary antibody. In each cell, the surface of LC3 dots was quantified and the phosphorylation of eIF2α was evaluated by measuring the cytoplasmic fluorescence intensity of the immunostaining. In untreated condition (Ctrl) (**A**), brefeldin A (**B**), PI-103 (**C**) and niclosamide (**D**)-treated cells, normalized GFP dots surface and peIF2α cytoplasmic intensity are depicted for 1500 randomly selected cells and the coefficient (R) for the correlation between both parameters was calculated using a Spearman’s rank test.

**Figure S3. Validation of U2OS RFP LC3 EIF2α^S51^ clones**

Human osteosarcoma U2OS cells stably expressing RFP-LC3 were gene edited with the CRISPR-Cas9 technique to establish an *EIF2a^S51A^* non-phosphorylable knockin mutant. Three sequence validated clones were selected (number 25, 59, 70) and subjected to further validation. For this, U2OS RFP-LC3 WT and the clones 25, 59, 70 were treated with arsenate at 1 mM for 1 h or thapsigargin at 3 μM for 6 h. After fixation, plates were stained with a phosphoneoepitope-specific eIF2α antibody followed by an AlexaFluor-488 secondary antibody. Nuclei were counterstained with Hoechst 33342 and eIF2α phosphorylation was assessed by fluorescence microscopy. Images were segmented and analyzed to determine the fluorescence intensity in the cytoplasm. Treatments were normalized as percent of untreated controls in each cell line. Mean ± SD of quadruplicates from one experiment are shown and statistical significance was analyzed using the Student’s-test. Differences to respective controls are depicted as *p < 0.05, **p < 0.01 ***p < 0.001.

**Figure S4. Validation of U2OS knockout for eIF2α kinases 1, 2, 3 and 4**

Human osteosarcoma U2OS cells stably expressing GFP-LC3 were modified with the CRISPR-Cas9 method to knockout each eIF2α kinase. For each of the four gene edits, one knockout clone was selected and further validated by immunoblot. To verify equal loading, β-actin (ACTB) was used (**A**). EIF2AK1 and EIF2AK3 mediated eIF2α phosphorylation in response to arsenate and thapsigargin treatments, respectively, was assessed. U2OS GFP-LC3 WT, *EIF2AK1^-/-^, EIF2AK2^-/-^, EIF2AK3^-/-^* and *EIF2AK4^-/-^* were treated with arsenate at 1 mM for 1 h or thapsigargin at 3 μM for 6 h and fixed, before staining with a phosphoneoepitope-specific eIF2α antibody followed by an AlexaFluor-488 secondary antibody. Nuclei were counterstained with Hoechst 33342 and eIF2α phosphorylation was assessed by fluorescence microscopy. The peIF2α fluorescence intensity in the cytoplasm was measured, data were normalized as percent of untreated controls in each cell line and. Mean ± SD of technical quadruplicates from one experiment are shown and statistical significance was analyzed using the Student’s-test. Differences to respective controls are depicted as *p < 0.05, **p < 0.01 ***p < 0.001 (**B**).

**Figure S5. Agents requiring eIF2α phosphorylation for complete autophagy induction**

The geometric distances of each point in LC3 dots surface between U2OS WT and *EIF2a^S51A^* (from **Fig. 3B, C, D**), as well as between MEF WT and knockout for *eif2ak1-4* (*4KO*) (**Fig. 4B**), was calculated. In U2OS, the mean of the distances of the three tested clones was calculated. Distances were subjected to a z-score transformation centered on control. Then the z-score of LC3 dots surface (from **Fig. 1C** for U2OS and **Fig. 2B** for MEF) as well as geometric distances were independently scaled between 0 and 1 with a sigmoidal transformation and represented. In addition to the torin 1 control, agents which are among the 40 % most potent autophagy inducers and for which autophagy depends on peIF2α (with a distance greater than 0.5) in both cell lines are shown.

**Table S1. Autophagy, eIF2α phosphorylation and distances**

The compounds of the custom arrayed library were used in **Fig. 1, 2, 3, 4, 5, 6, S1 and S4**at the indicated concentrations. Autophagy (AG), eIF2α phosphorylation (peIF2α) and geometric distance (dist) are depicted as average raw values (.Av), as z-score (.sca) or normalized as percent of induction (.Norm). In order to build heatmaps data was subjected to sigmoidal transformation (.HM).

**Table S2. Autophagy and eIF2**α **phosphorylation related to figure 7F**

Guanabenz, nelfinavir, salubrinal, sephin 1 and torin 1 were used in **Fig. 7F** at the indicated concentrations. Autophagy (AG) and eIF2α phosphorylation (peIF2α) values were scaled with a z-score transformation (.sca).
