# Supplementary figures and images for: Phosphorylation of eukaryotic initiation factor-2α (eIF2α) in autophagy
Source: Cell Death Dis. 2020 Jun 8;11(6):433. doi: 10.1038/s41419-020-2642-6 (PMC7280501; doi:10.1038/s41419-020-2642-6)

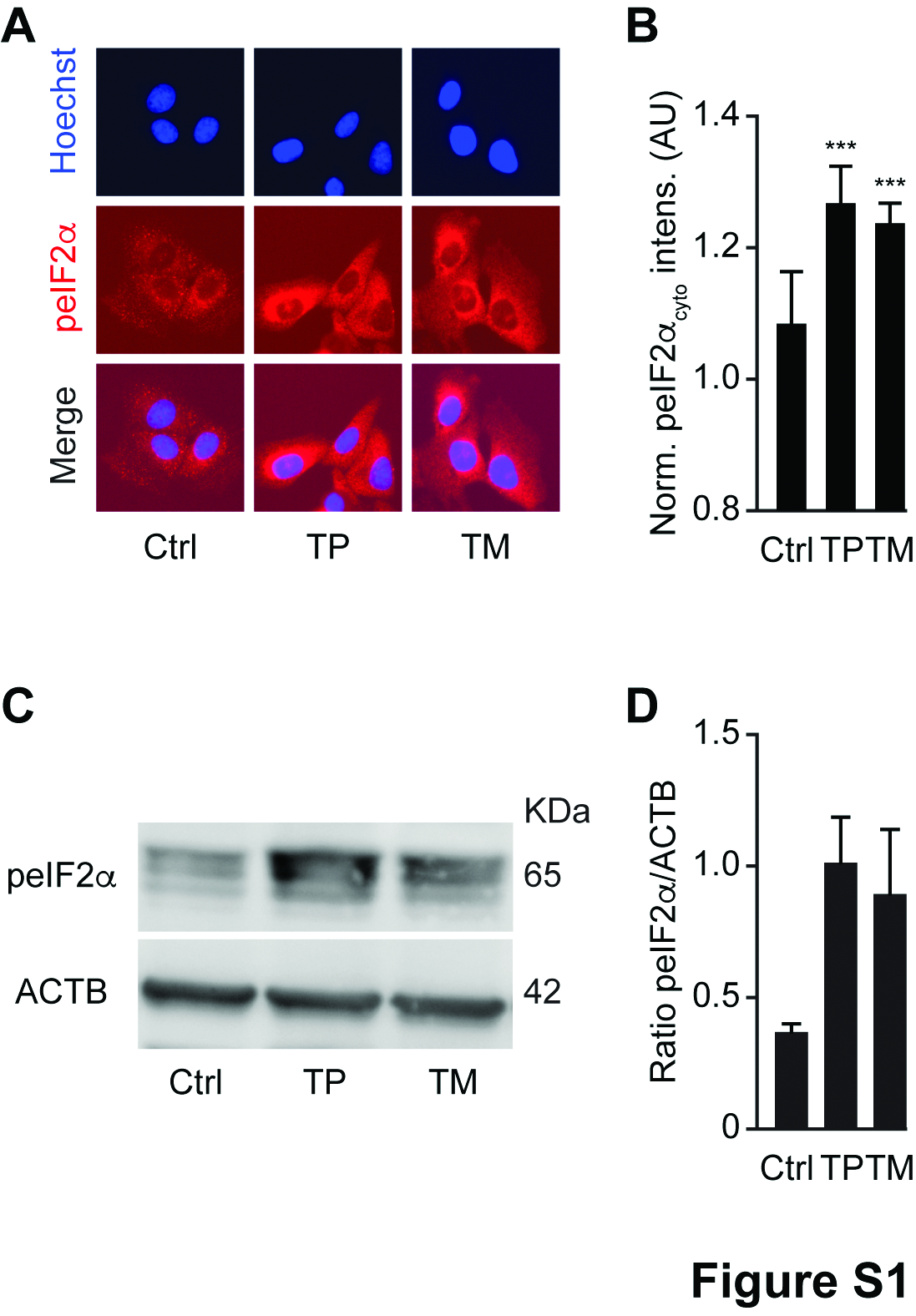

Supplement: Supplementary file 2 — Figure S1. Validation of eIF2αS51 antibody for U2OS cells [file 41419_2020_2642_MOESM2_ESM.tif]

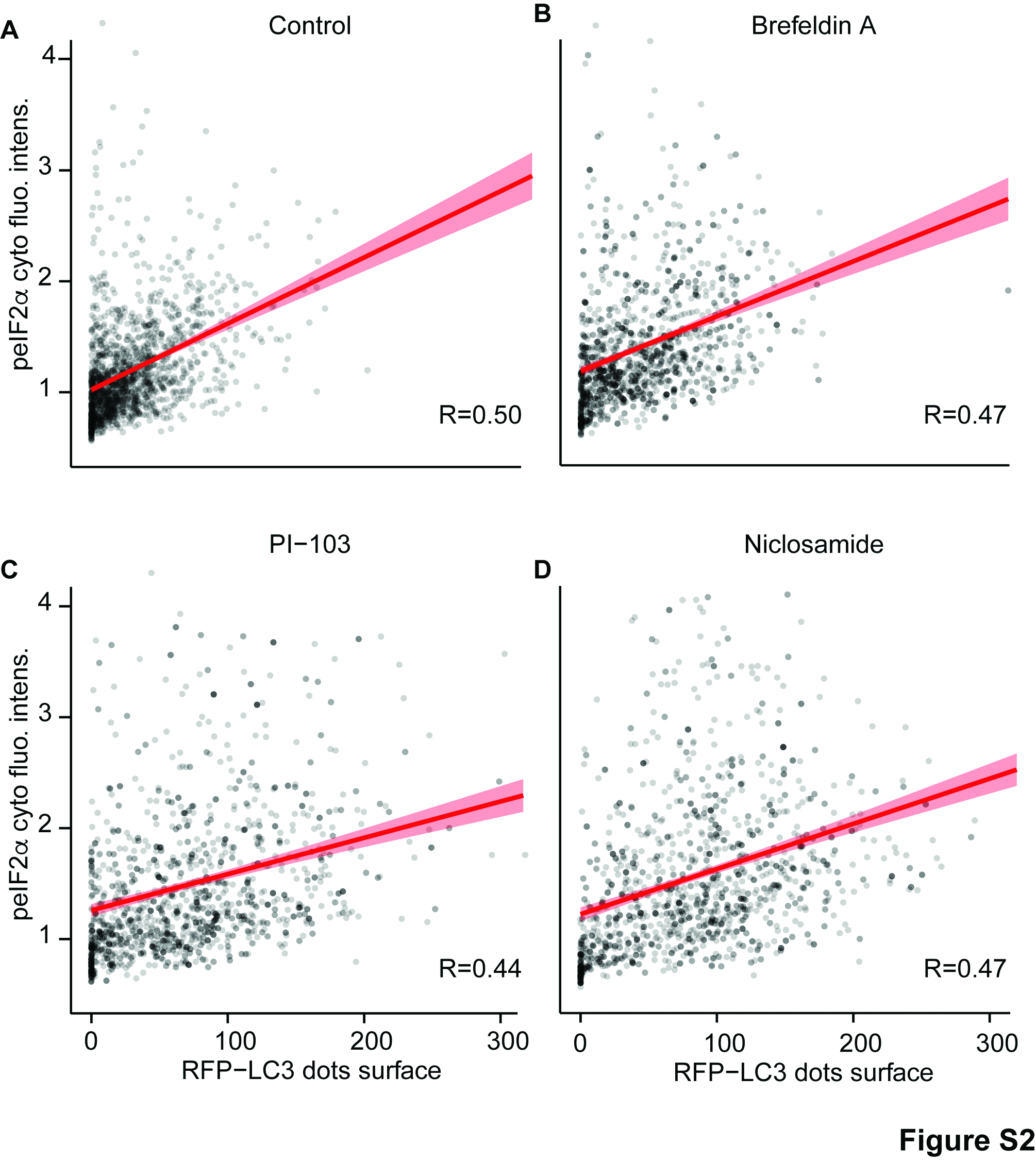

Supplement: Supplementary file 3 — Figure S2. Correlation between autophagy and peIF2α in a cell-per-cell basis [file 41419_2020_2642_MOESM3_ESM.tif]

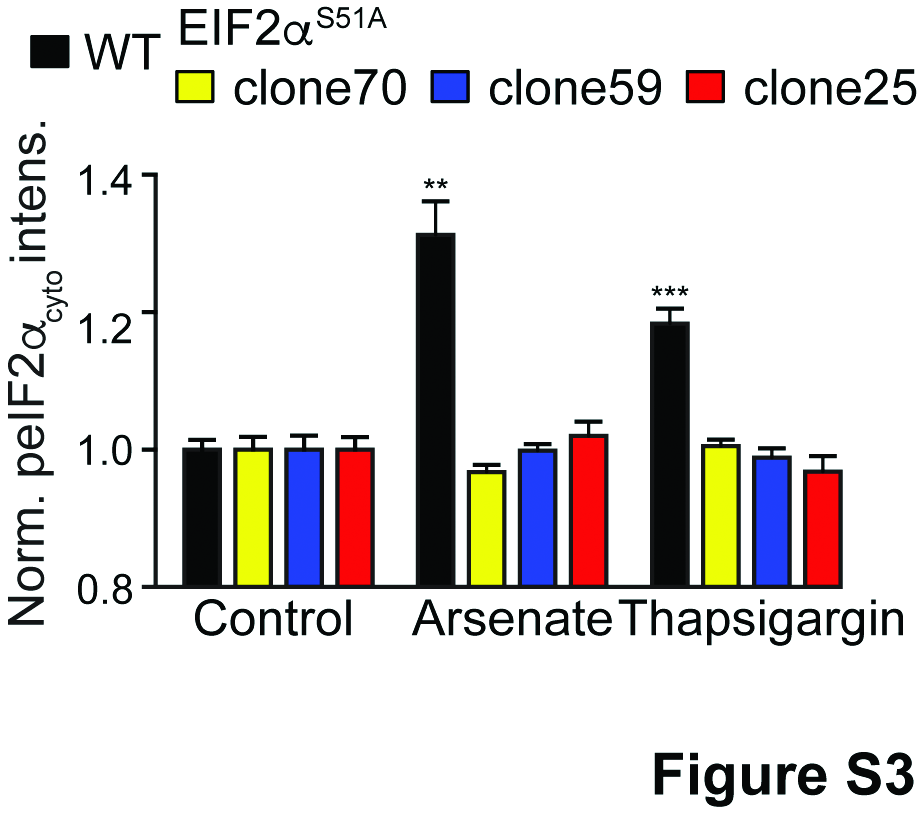

Supplement: Supplementary file 4 — Figure S3. Validation of U2OS RFP LC3 EIF2αS51 clones [file 41419_2020_2642_MOESM4_ESM.tif]

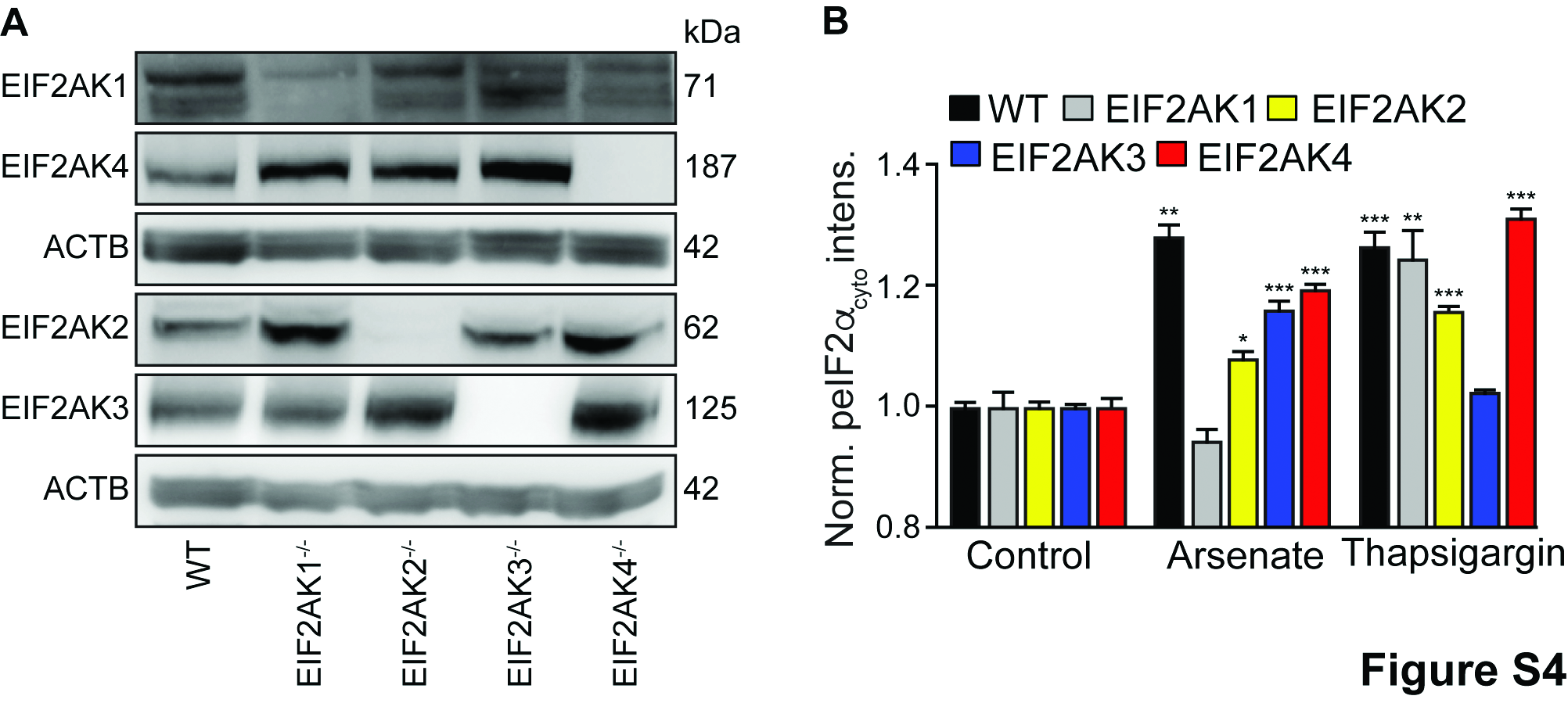

Supplement: Supplementary file 5 — Figure S4. Validation of U2OS knockout for eIF2α kinases 1, 2, 3 and 4 [file 41419_2020_2642_MOESM5_ESM.tif]

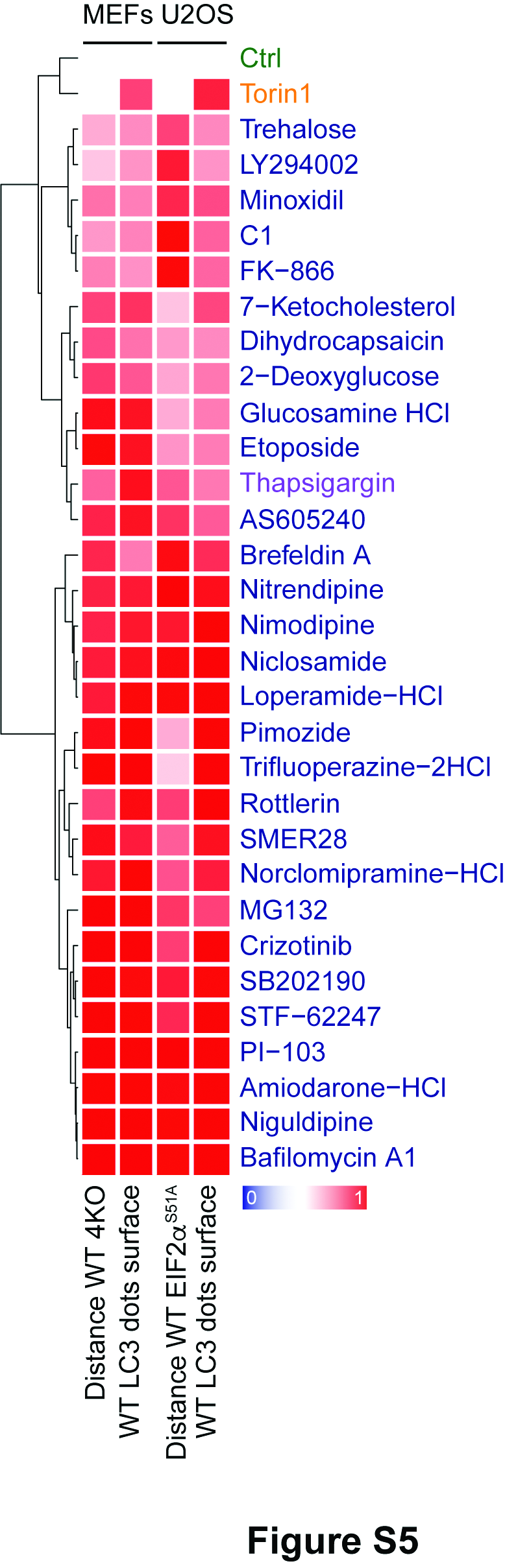

Supplement: Supplementary file 6 — Figure S5. Agents requiring eIF2α phosphorylation for complete autophagy induction [file 41419_2020_2642_MOESM6_ESM.tif]
